# In Gastric Cancer Patients Receiving Neoadjuvant Chemotherapy Systemic Inflammation Response Index is a Useful Prognostic Indicator

**DOI:** 10.3389/pore.2021.1609811

**Published:** 2021-10-12

**Authors:** Li Chen, Yong Chen, Lele Zhang, Yingwei Xue, Shiwei Zhang, Xingrui Li, Hongjiang Song

**Affiliations:** ^1^ Department of Thyroid and Breast Surgery, Tongji Hospital, Tongji Medical College, Huazhong University of Science and Technology, Wuhan, China; ^2^ Department of Gastrointestinal Surgery, Harbin Medical University Cancer Hospital, Harbin Medical University, Harbin, China; ^3^ Department of General Surgery, Huai’an Second People’s Hospital and the Affiliated Huai’an Hospital of Xuzhou Medical University, Huai’an, China; ^4^ Department of Oncology Surgery, The First People’s Hospital of Fuyang Hangzhou, Hangzhou, China

**Keywords:** prognosis, neoadjuvant chemotherapy, advanced gastric cancer, systemic inflammation response index (SIRI), tumor indicator

## Abstract

**Background:** The preoperative systemic inflammation response index (SIRI), based on peripheral neutrophil (N), monocyte (M), and lymphocyte (L) counts, has shown mounting evidence as an effective prognostic indicator in some malignant tumors. The aim of the present study was to evaluate the prognostic significance of pre-treatment SIRI in gastric cancer patients who received neoadjuvant chemotherapy (NACT).

**Methods:** This retrospective study comprised 107 patients with advanced gastric cancer treated with NACT between July 2007 and September 2015 in our hospital. SIRI was calculated from peripheral venous blood samples obtained prior to treatment. The best cutoff value for SIRI by receiver operating characteristic (ROC) curve was 1.2 (low SIRI <1.21, high SIRI ≥1.21). The clinical outcomes of disease-free survival (DFS) and overall survival (OS) were analyzed by Kaplan-Meier survival analysis and compared using the log-rank test. Univariate and multivariate analyses were performed by the Cox proportional hazards regression model.

**Results:** The results demonstrated that the low SIRI group was statistically associated with gender, primary tumor site, white blood cell, neutrophil, and monocyte counts, NLR (neutrophil to lymphocyte ratio), MLR (monocyte to lymphocyte ratio), and PLR (platelet to lymphocyte ratio). The SIRI was predictive for DFS and OS by univariate and multivariate analysis; the low SIRI group had better median DFS and OS than the high SIRI group (median DFS 27.03 *vs*. 22.33 months, median OS 29.73 *vs*. 24.43 months). The DFS and OS in the low SIRI group were longer than the high SIRI group.

**Conclusions:** SIRI may qualify as a useful, reliable, and convenient prognostic indicator in patients with advanced gastric cancer to help physicians to provide personalized prognostication for gastric cancer patients treated with NACT.

## Introduction

Globally, gastric cancer (GC) is the sixth most common cancer and the third leading cause of cancer-related death; clearly, it remains a critical public health problem (1). According to worldwide statistics from 2020, a total of 1,089,000 new cases were diagnosed, and about 769,000 cases resulted in death in which many cases had advanced-stage disease at the time of diagnosis (2). Although the incidence and mortality rates of gastric cancer have declined over the last few decades in most parts of the world, the occurrence of the diffuse type of gastric cancer has increased, and a trend to a younger age of onset has been reported (3). Moreover, the prognosis of gastric cancer remains poor, with an average five-year survival rate of less than 30% and a median overall survival of less than 1 year (4). Complete surgical resection continues to form the basis of treatment, and the rate of surgical resection can be improved with the addition of radiotherapy and chemotherapy (5). Neoadjuvant chemotherapy (NACT), like in other malignancies, now plays an important role in the treatment of gastric cancer (6). NACT has increased overall survival in gastric cancer and improved the pathological complete response rate (7).

Inflammation has been identified as a critically important factor in carcinogenesis, and systemic inflammatory cells likely play important roles in the development, progression, and metastasis of malignant tumors (8). These cells, including neutrophils, monocytes, platelets, and lymphocytes, may present potential immuno-therapeutic targets in gastric cancer, as well as yielding prognostic information. Our understanding of the full role of these cells as well as their relative interaction, such as NLR (neutrophil to lymphocyte ratio), MLR (monocyte to lymphocyte ratio), and PLR (platelet to lymphocyte ratio), remains incomplete; these systemic inflammatory response markers have been widely used to determine the prognosis of tumors (9-11).

In recent years, the preoperative systemic inflammation response index (SIRI), based on peripheral neutrophil, monocyte, and lymphocyte counts, has gained credibility as an effective prognostic indicator in some malignancies (12, 13). However, the SIRI has rarely been studied in patients with advanced gastric cancer who received NACT. Moreover, the efficacy of systemic inflammatory response markers to help predict which patients would benefit from specific neoadjuvant chemotherapeutic agents must be considered. Therefore, the present study intended to investigate the prognostic significance of the preoperative SIRI in patients with advanced gastric cancer undergoing NACT.

## Materials and Methods

### Patient Selection

This retrospective study comprised 107 gastric cancer patients, enrolled at our hospital from July 2007 to September 2015, who were treated with neoadjuvant chemotherapy. All cancers were histologically confirmed, and clinical data were extracted from the medical records. Our study was approved by the ethics committee of Harbin Medical University Cancer Hospital, and informed consent was obtained from all enrolled patients.

The inclusion criteria included the following (1): patients with advanced histologically-confirmed gastric cancer (excluding distant metastasis) (2); surgical treatment (3); overall survival time ≥3 months (4); no cancer treatment, such as chemotherapy or radiotherapy, prior to evaluation at our hospital; and (5) complete clinical data and postoperative follow-up. The exclusion criteria included the following (1): co-existing malignancies (2); surgical complications or infection (3); co-existing inflammatory or autoimmune disease; and (4) patients who had received blood product transfusion or peripheral blood tests were not available.

### Treatment Methods for NACT

All enrolled patients received preoperative NACT, and the standard regimens for advanced gastric cancer included SOX and XELOX regimens. For the SOX regimen, Oxaliplatin was 130 mg/ m^2^ on the first day and S-1 60 mg twice daily for 2 weeks. For the XELOX regimen, Oxaliplatin 130 mg/ m^2^ on the first day and Capecitabine 1,500 mg twice daily for 2 weeks. Cycles were repeated every 3 weeks.

### Evaluation of Response

The TNM stage system was used as per the eighth edition of the Union for International Cancer Control and the American Joint Committee on Cancer TNM stage classification (14). Response Evaluation Criteria in Solid Tumors (RECIST) guidelines was performed to evaluate the response (15), and included the following categories: 1) complete response (CR): all target lesions disappeared; 2) partial response (PR): the sum of the longest diameters of target lesions was decreased at least 30%; 3) stable disease (SD): between PR and PD; and 4) progression of disease (PD): the sum of the longest diameters of target lesions was increased by at least 20%, or one or more new lesions appeared. The toxicity of NACT was determined by the National Cancer Institute Common Toxicity Criteria (NCI-CTC) (16).

### Peripheral Venous Blood Parameters

Peripheral venous blood was collected at defined points of time prior to NACT. All samples were collected into EDTA anticoagulant tubes and obtained whilst fasting. The SIRI was defined as follows: SIRI = N × M/L (the units were N (10^9^/L), M (10^9^/L), and L (10^9^/L)) where N, M, and L are pretreatment peripheral neutrophil (N), monocyte (M), and lymphocyte (L) counts, respectively.

### Follow up

All enrolled patients were followed regularly by telephone or as inpatients and outpatients. The postoperative schedule was every 3 months for the first and second years, every 6 months for the third through the fifth years, and then at 12-months intervals thereafter. Disease-free survival (DFS) was defined as the interval from the surgical date to relapse (local recurrence or distant metastases). Overall survival (OS) was defined as the interval from the surgical date to death from any cause or last follow-up.

### Statistical Analysis

All statistical analyses were performed using SPSS software 17.0 (Chicago, IL, United States) and GraphPad prism software 8.0 (La Jolla, CA, United States). The best cutoff value for SIRI was determined by ROC analysis. The Chi-square test and Fisher’s exact test were used to analyze the relationship between SIRI and clinicopathological features. The clinical outcomes of DFS and OS were analyzed by Kaplan-Meier survival analysis and compared using the log-rank test. Univariate and multivariate analyses were performed by the Cox proportional hazards regression model. A two-tailed *p* < 0.05 was considered statistically significant.

## Results

### Demographic and Clinicopathological Characteristics

The demographic and clinicopathological characteristics of the 107 patients are shown in Table 1. There were 82 males and 25 females, with an age range from 32 to 73 years (median, 56 years). The best cutoff value by ROC analysis for pre-treatment SIRI was 1.21; 62 patients (57.9%) in the low SIRI group and 45 patients (42.1%) in the high SIRI group. Compared to the high SIRI group, the low SIRI group was significantly associated with gender (χ^2^ = 12.090, *p* < 0.001) and primary tumor site (χ^2^ = 6.237, *p* = 0.047). (Table 1).

**TABLE 1:** Baseline clinicopathological characteristics of all enrolled patients.

Parameters		Low SIRI <1.21	High SIRI ≥1.21	χ^2^	P Value
Cases (n)	Number (%)	62	45		
Age (years)				0.116	0.733
<56	52 (48.6%)	31 (50.0%)	21 (46.7%)		
≥56	55 (51.4%)	31 (50.0%)	24 (53.3%)		
Gender					<0.001^a^
Male	82 (76.6%)	40 (64.5%)	42 (93.3%)		
Female	25 (23.4%)	22 (35.5%)	3 (6.7%)		
BMI				2.823	0.093
<22.10	53 (49.5%)	35 (56.5%)	18 (40.0%)		
≥22.10	54 (50.5%)	27 (43.5%)	27 (60.0%)		
ABO blood type				2.337	0.523^a^
A	30 (28.0%)	17 (27.4%)	13 (28.9%)		
B	36 (33.6%)	19 (30.6%)	17 (37.8%)		
O	31 (29.0%)	18 (29.1%)	13 (28.9%)		
AB	10 (9.4%)	8 (12.9%)	2 (4.4%)		
Radical resection				5.331	0.070
R0	60 (56.1%)	38 (61.3%)	22 (48.9%)		
R1	24 (22.4%)	9 (14.5%)	15 (33.3%)		
R2	23 (21.5%)	15 (24.2%)	8 (17.8%)		
Type of surgery				2.708	0.243^a^
distal gastrectomy	60 (56.1%)	35 (56.5%)	25 (55.6%)		
proximal gastrectomy	7 (6.5%)	2 (3.2%)	5 (11.1%)		
total gastrectomy	40 (37.4%)	25 (40.3%)	15 (33.3%)		
Differentiation				3.587	0.168^a^
poorly differentiated	65 (60.8%)	42 (67.8%)	23 (51.1%)		
moderately differentiated	36 (33.6%)	18 (29.0%)	18 (40.0%)		
well differentiated	6 (5.6%)	2 (3.2%)	4 (8.9%)		
Primary tumor site				6.237	0.047^a^
upper 1/3	9 (8.4%)	2 (3.2%)	7 (15.6%)		
middle 1/3	39 (36.5%)	23 (37.1%)	16 (35.6%)		
low 1/3	59 (55.1%)	37 (59.7%)	22 (48.8%)		
Pathology				2.311	0.467^a^
normal (Tis)	9 (8.4%)	7 (11.3%)	2 (4.4%)		
Adenocarcinoma	61 (57.0%)	33 (53.2%)	28 (62.2%)		
mucinous carcinoma	8 (7.5%)	5 (8.1%)	3 (6.7%)		
signet ring cell carcinoma	12 (11.2%)	9 (14.5%)	3 (6.7%)		
mixed carcinoma	17 (15.9%)	8 (12.9%)	9 (20.0%)		
Clinical TNM classification					
T stage				2.538	0.206^a^
T3	9 (8.4%)	5 (8.1%)	4 (8.9%)		
T4a	68 (63.6%)	36 (58.1%)	32 (7.1%)		
T4b	30 (28.0%)	21 (33.9%)	9 (20.0%)		
N stage				1.145	0.551^a^
N0	30 (28.0%)	16 (25.8%)	14 (31.1%)		
N1	72 (67.3%)	42 (67.7%)	30 (66.7%)		
N2	5 (4.7%)	4 (6.5%)	1 (2.2%)		
TNM stage				1.231	0.267
II	23 (21.5%)	11 (17.7%)	12 (26.7%)		
III	84 (78.5%)	51 (82.3%)	33 (73.3%)		
Pathological TNM classification					
T stage				4.201	0.299^a^
Tis + T1	17 (15.9%)	11 (17.7%)	6 (13.3%)		
T2	3 (2.8%)	3 (4.8%)	0 (0.0%)		
T3	42 (39.3%)	20 (32.3%)	22 (48.9%)		
T4a	21 (19.6%)	15 (24.2%)	6 (13.3%)		
T4b	24 (22.4%)	13 (21.0%)	11 (24.4%)		
N stage				2.938	0.421^a^
N0	32 (29.9%)	21 (33.9%)	11 (24.4%)		
N1	23 (21.5%)	15 (24.2%)	8 (17.8%)		
N2	18 (16.8%)	8 (12.9%)	10 (22.2%)		
N3a	25 (23.4%)	13 (21.0%)	12 (26.7%)		
N3b	9 (8.4%)	5 (8.0%)	4 (8.9%)		
Metastasis				0.096	0.756^a^
M0	104 (97.2%)	60 (96.8%)	44 (97.8%)		
M1	3 (2.8%)	2 (3.2%)	1 (2.2%)		
TNM stage				2.137	0.551^a^
Tis + I	15 (14.0%)	11 (17.8%)	4 (8.9%)		
II	31 (29.0%)	16 (25.8%)	15 (33.3%)		
III	58 (54.2%)	33 (53.2%)	25 (55.6%)		
IV	3 (2.8%)	2 (3.2%)	1 (2.2%)		
Total lymph nodes				1.127	0.288
<27	53 (49.5%)	28 (45.2%)	25 (55.6%)		
≥27	54 (50.5%)	34 (54.8%)	20 (44.4%)		
Positive lymph nodes				1.670	0.434
0	33 (30.8%)	21 (33.9%)	12 (26.7%)		
<3	20 (18.7%)	13 (21.0%)	7 (15.6%)		
≥3	54 (50.5%)	28 (45.1%)	26 (57.7%)		
Lauren classification				0.363	0.834
Intestinal	57 (53.3%)	33 (53.2%)	24 (53.3%)		
Diffuse	31 (29.0%)	19 (30.7%)	12 (26.7%)		
Mixed	19 (17.8%)	10 (16.1%)	9 (20.0%)		
Borrmann classification				1.122	0.925^a^
Borrmann I	1 (0.9%)	1 (1.6%)	0 (0.0%)		
Borrmann II	24 (22.4%)	15 (24.2%)	9 (20.0%)		
Borrmann III	67 (62.6%)	37 (59.7%)	30 (66.7%)		
Borrmann IV	15 (14.0%)	9 (14.5%)	6 (13.3%)		
Tumor size (mm)				0.047	0.829
<50	56 (52.3%)	33 (53.2%)	23 (51.1%)		
≥50	51 (47.7%)	29 (46.8%)	22 (48.9%)		

a Performed using the Fisher’s exact test.

### Blood Parameters

We analyzed blood parameters by median value (Table 2). Compared with the high SIRI group, the low SIRI group was significantly associated with white blood cell (χ2 = 27.100, *p* < 0.001)、N (χ^2^ = 35.866, *p* < 0.001)、M (χ2 = 21.628, *p* < 0.001)、NLR (χ2 = 51.321, *p* < 0.001)、MLR (χ2 = 45.862, *p* < 0.001) and PLR (χ2 = 4.293, *p* < 0.05), however, the low SIRI group was not associated with hemoglobin (χ^2^ = 1.001, *p* > 0.05)、*p* (χ^2^ = 0.116, *p* > 0.05)、or L (χ^2^ = 0.449, *p* > 0.05). (Table 2).

**TABLE 2:** Correlation between SIRI and hematological parameters.

Parameters	Low SIRI<1.21	High SIRI≥1.21	χ^2^	P Value
Cases (n)	62	45		
White blood cell (×10^9^/L)			27.100	<0.001
<6.42	44 (71.0%)	9 (20.0%)		
≥6.42	18 (29.0%)	36 (80.0%)		
Hemoglobin (×10^9^ /L)			1.001	0.317
<121	27 (43.5%)	24 (53.3%)		
≥121	35 (56.5%)	21 (46.7%)		
Neutrophils (×10^9^ /L)			35.866	<0.001
<3.82	46 (74.2%)	7 (15.6%)		
≥3.82	16 (25.8%)	38 (84.4%)		
Monocyte (×10^9^ /L)			21.628	<0.001
<0.45	42 (67.7%)	10 (22.2%)		
≥0.45	20 (32.3%)	35 (77.8%)		
Platelet (×10^9^ /L)			0.116	0.733
<285	31 (50.0%)	21 (46.7%)		
≥285	31 (50.0%)	24 (53.3%)		
Lymphocyte (×10^9^ /L)			0.449	0.503
<1.72	29 (46.8%)	24 (53.3%)		
≥1.72	33 (53.2%)	21 (46.7%)		
NLR			51.321	<0.001^a^
<2.18	49 (79.0%)	4 (8.9%)		
≥2.18	13 (21.0%)	41 (91.1%)		
MLR			45.862	<0.001
<0.28	48 (77.4%)	5 (11.1%)		
≥0.28	14 (22.6%)	40 (88.9%)		
PLR			4.293	0.038
<164	36 (58.1%)	17 (37.8%)		
≥164	26 (41.9%)	28 (62.2%)		

a Performed using the Fisher’s exact test.

### Correlation Between SIRI and Chemotherapy

All patients were received neoadjuvant chemotherapy, and the results indicated that the SIRI was associated with NACT regimens (χ^2^ = 7.151, *p* = 0.028). After the operation, 93 patients were received postoperative chemotherapy, and the results have shown that SIRI was not associated with postoperative chemotherapy regimens (χ^2^ = 2.103, *p* = 0.717). (Table 3).

**TABLE 3:** Correlation between SIRI and chemotherapy.

Parameters		Low SIRI <1.21	High SIRI ≥1.21	χ2	P Value
Cases (n)	Number (%)	62	45		
NACT regimens					
SOX	35 (32.7%)	17 (27.4%)	18 (40.0%)	7.151	0.028
XELOX	56 (52.3%)	39 (62.9%)	17 (37.8%)		
Others^a^	16 (15.0%)	6 (9.7%)	10 (22.2%)		
Preoperative chemotherapy times					
<3	57 (53.3%)	38 (61.3%)	19 (42.2%)	3.809	0.051
≥3	50 (46.7%)	24 (38.7%)	26 (57.8%)		
Postoperative chemotherapy regimens					
SOX	32 (29.9%)	16 (25.8%)	16 (35.5%)	2.103	0.717
XELOX	41 (38.3%)	26 (41.9%)	15 (33.3%)		
Others^a^	20 (18.7%)	13 (21.0%)	7 (15.6%)		
No	14 (13.1%)	7 (11.3%)	7 (15.6%)		
Postoperative chemotherapy times					
0	14 (13.1%)	7 (11.3%)	7 (15.6%)	2.198	0.333
<4	51 (47.7%)	27 (43.5%)	24 (53.3%)		
≥4	42 (39.2%)	28 (45.2%)	14 (31.1%)		
Response					
CR	9 (8.4%)	7 (11.3%)	2 (4.4%)	7.488	0.112^b^
PR	72 (67.3%)	45 (72.6%)	27 (60.0%)		
SD	7 (6.5%)	4 (6.4%)	3 (6.7%)		
PD	19 (17.8%)	6 (9.7%)	13 (28.9%)		

a DCF, docetaxel, cisplatin, and fluorouracil and other fluoropyrimidine-based adjuvant chemotherapy; ECF, epirubicin, cisplatin, and fluorouracil; FOLFOX, folinic acid, oxaliplatin, and fluorouracil; TCF, paclitaxel, cisplatin, and fluorouracil; TF, docetaxel and fluorouracil; TS, paclitaxel and S-1.

b Performed using the Fisher’s exact test.

### Univariate and Multivariate Cox Regression Survival Analyses

**FIGURE 1:**
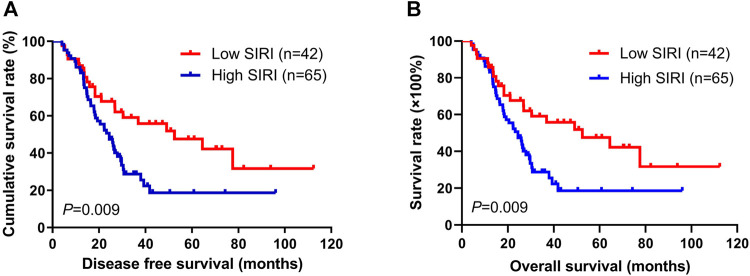
Kaplan-Meier analysis of DFS and OS for the SIRI of all patients with advanced gastric cancer.

Using the best cutoff value of 1.21 for the SIRI, it significantly correlated with DFS and OS. In univariate analysis, low SIRI was correlated with prolonged DFS and OS (hazard ratio (HR): 3.437, 95% confidence interval (CI): 1.059-11.149, *p* = 0.009; HR: 3.331, 95% CI: 1.001-11.082, *p* = 0.021). In multivariate analysis, low SIRI was also correlated with prolonged DFS and OS (HR: 1.782, 95% CI: 1.241-3.942, *p* = 0.024; HR: 1.665, 95% CI: 1.302-3.613, *p* = 0.028; Supplementary Table S1). Kaplan-Meier survival curves for DFS and OS for the SIRI of all patients are shown in Figure 1.

### Survival and Evaluation of SIRI

The 1-year, 3-years, and 5-years survival rates of DFS and OS in the low SIRI group were 71.0% (44/62), 30.6% (19/62), and 14.5% (9/62); and 85.5% (53/62), 32.3% (20/62), and 19.4% (12/62), respectively. The 1-year, 3-years, and 5-years survival rates of DFS and OS in the high SIRI group were 75.6% (34/45), 15.6% (7/45), and 2.2% (1/45); and 84.4% (38/45), 17.8% (8/45), and 2.2% (1/45), respectively (Table 4). Notably, patients in the high SIRI group had worse 3-years DFS and OS than the low SIRI group (χ2 = 3.228, *p* = 0.072; χ2 = 2.830, *p* = 0.093) and worse 5-years DFS and OS than the low SIRI group (χ2 = 4.651, *p* = 0.031; χ2 = 7.171, *p* = 0.007).

**TABLE 4:** 1-year, 3-years, and 5-years DFS and OS rates of patients with advanced gastric cancer.

Parameters	Case (n)	1-year (%)	DFS			OS	
			3-years (%)	5-years (%)	1-year (%)	3-years (%)	5-years (%)
Low SIRI	62 (59.8%)	44 (71.0%)	19 (30.6%)	9 (14.5%)	53 (85.5%)	20 (32.3%)	12 (19.4%)
High SIRI	45 (40.2%)	34 (75.6%)	7 (15.6%)	1 (2.2%)	38 (84.4%)	8 (17.8%)	1 (2.2%)
χ^2^		0.278	3.228	4.651	0.022	2.830	7.171
*P* value		0.598	0.072	0.031^#^	0.882	0.093	0.007^#^

### Association of SIRI and Borrmann Classification

**FIGURE 2:**
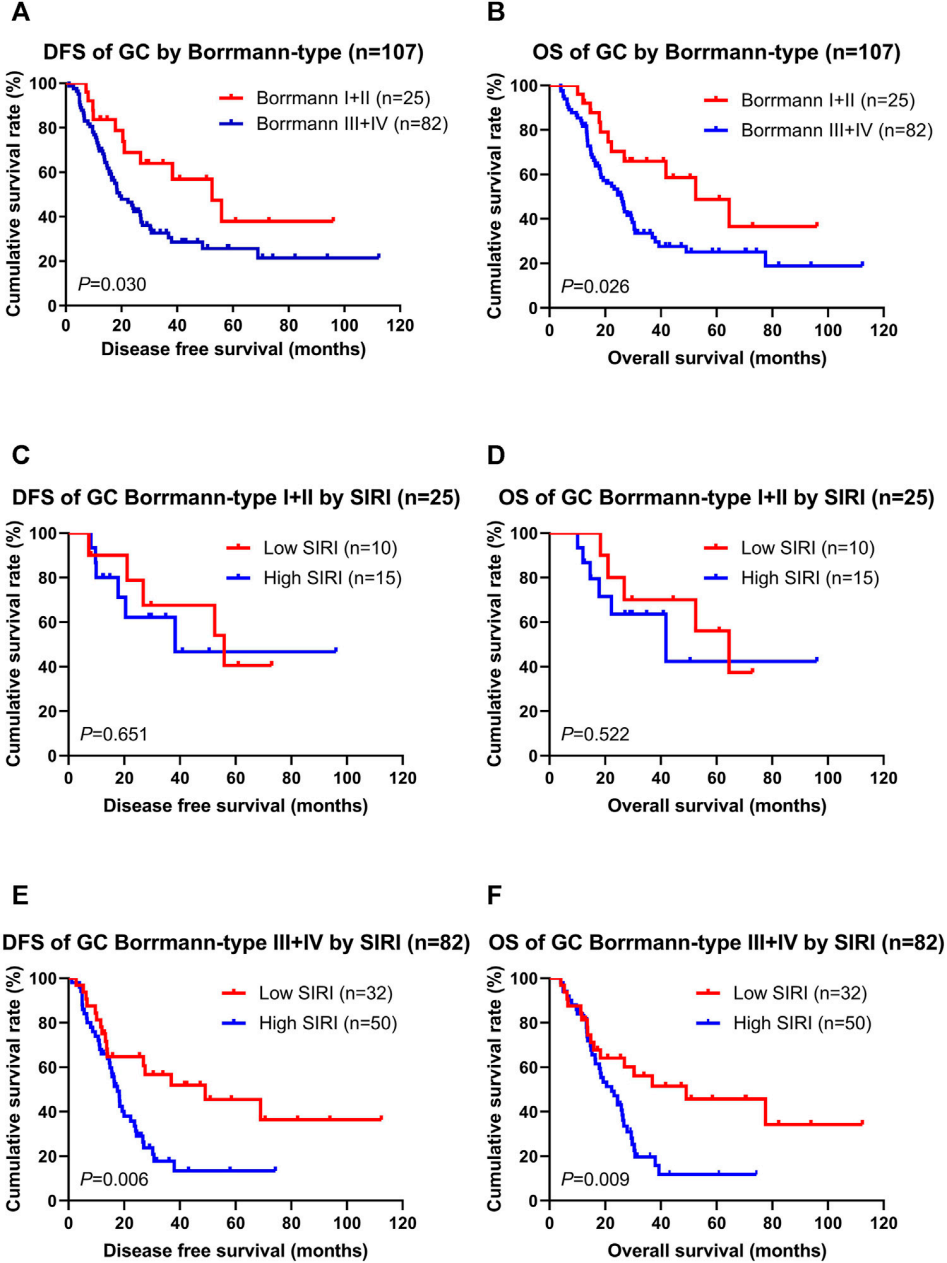
DFS and OS for the SIRI of patients with gastric cancer in different Borrmann classification. Borrmann I + II group means patients with Borrmann I or Borrmann II (25 patients); Borrmann III + IV group means patients with Borrmann III or Borrmann IV (82 patients).

According to multivariate Cox regression model analyses, the Borrmann classification was a significant prognostic factor (Supplementary Table S1). We stratified into two groups, Borrmann I + II and Borrmann III + IV. The results showed that patients with Borrmann I + II had longer DFS and OS than Borrmann III + IV (χ^2^ = 4.690, *p* = 0.030; χ^2^ = 4.986, *p* = 0.026; Figures 2A,B). Moreover, the low SIRI group had longer DFS and OS than the high SIRI group in Borrmann I + II (χ^2^ = 0.204, *p* = 0.651; χ2 = 0.410, *p* = 0.522; Figures 2C,D), and longer DFS and OS than the high SIRI group in Borrmann III + IV (χ^2^ = 7.434, *p* = 0.006 and χ2 = 6.884, *p* = 0.009, respectively; Figures 2E,F).

### Association of Pathologic Stage and SIRI

**FIGURE 3:**
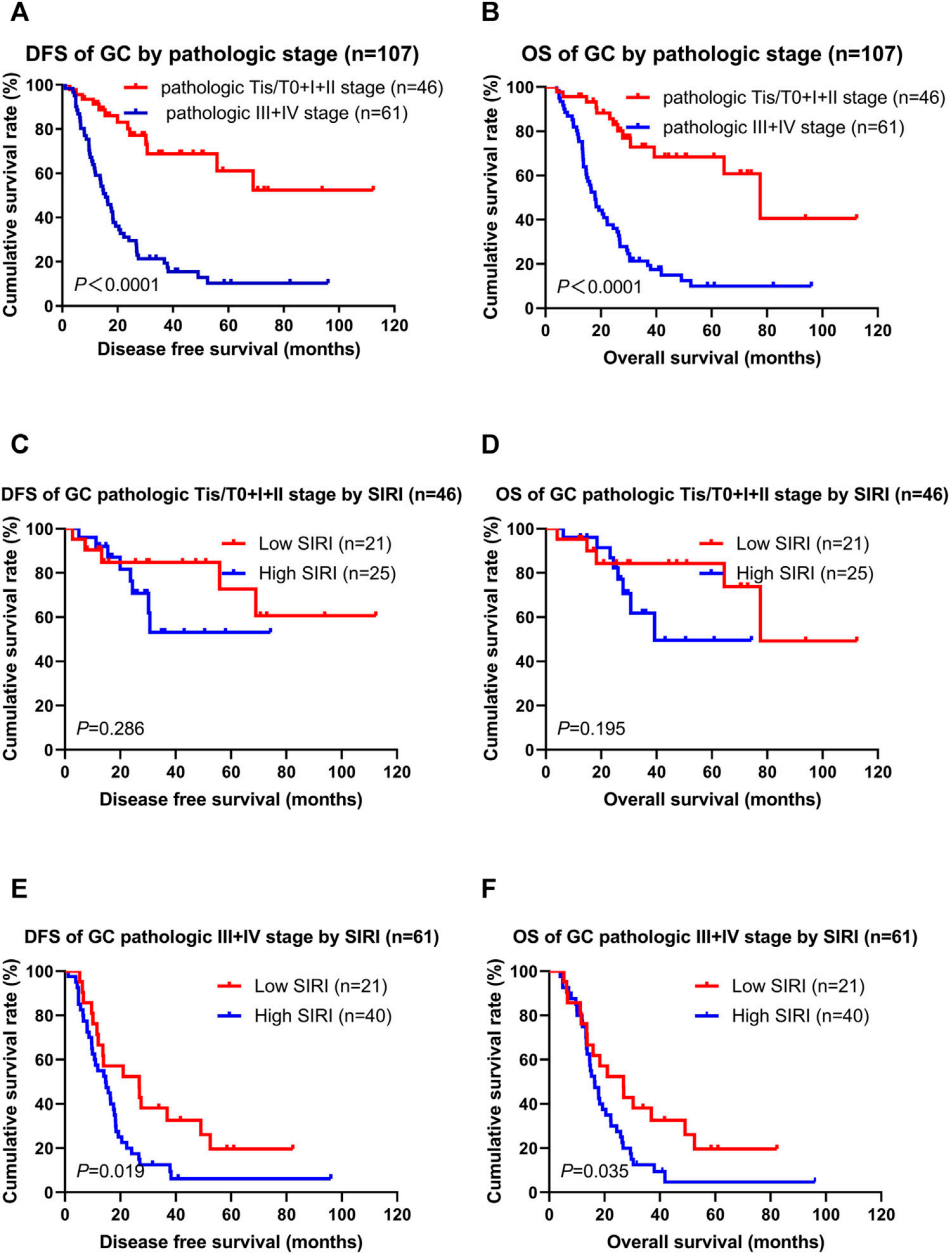
DFS and OS for the SIRI of patients with gastric cancer in different pathologic stages. Pathologic Tis/T0+I + II stage means patients with pathologic Tis/T0 or I or II stages (46 patients); pathologic III + IV stage means patients with pathologic III or IV stages (61 patients).

Not surprisingly, patients with the pathologic Tis/T0 + I + II stages had longer DFS and OS than the pathologic III + IV stages (χ^2^ = 28.850, *p* < 0.0001; χ^2^ = 31.030, *p* < 0.0001; Figures 3A,B). The low SIRI group had longer DFS and OS than the high SIRI group in the pathologic Tis/T0 + I + II stages (χ^2^ = 1.137, *p* = 0.286; χ^2^ = 1.683, *p* = 0.195; Figures 3C,D), and longer DFS and OS than the high SIRI group in the pathologic III + IV stages (χ^2^ = 5.503, *p* = 0.019; χ2 = 4.431, *p* = 0.035; Figures 3E,F).

### Correlation Between SIRI and Toxicity Assessment

NACT toxicity was evaluated following two treatment cycles. The most common toxicities were hematologic. There was no correlation with SIRI relative to anemia, leucopenia, neutropenia, myelosuppression, or gastrointestinal reaction (*p* > 0.05), but it did correlate with thrombocytopenia (*p* < 0.05).

## Discussion

Despite advances in surgical techniques and adjuvant therapy in recent decades, gastric cancer overall continues to be associated with a poor outcome, rapid recurrence, and metastasis (17). NACT has evolved into a critical part of gastric carcinoma treatment, importantly without increasing postoperative complications (18). Therefore, in order to optimize chemotherapy selection, accurate prognostic indicators that have been assessed in patients treated with NACT are most valuable.

Inflammation, as an important component of the tumor microenvironment (TME), is related to tumor development and progression (19). Systemic inflammation is an ancestral physiological response, and tumor cells influence proinflammatory mediators (20, 21). Increasingly, studies have provided convincing evidence that systemic inflammatory cells are associated with prognosis in many malignancies (22-24). Cellular components of the systemic inflammatory response may reflect and predict survival for gastric cancer. Moreover, inflammation and immune-based biomarkers, such as NLR, MLR, and PLR, have been statistically validated as prognostic markers (25-27). The combined cellular elements of SIRI provide a comprehensive balance of host immune and inflammatory status, reported to be associated with survival times and clinical outcomes. To date, however, there is no evidence whether SIRI can serve as a useful indicator to predict outcomes of gastric cancer patients that have received NACT.

In this study, the SIRI was a significant prognostic factor that could predict prognosis and treatment response. Low SIRI correlated well with DFS and OS. Therefore, SIRI appears to be a cost-effective, convenient, noninvasive, and reproducible biomarker for treatment response in patients with advanced gastric cancer treated with NACT and surgical resection.

Cancer-related inflammation is considered the seventh hallmark of cancer and several potential mechanisms might explain why a low SIRI was associated with better survival than the high SIRI group. Neutrophils can inhibit the immune system by releasing cytokines and chemokines, promote circulating tumor cells (CTCs), and stimulate tumor angiogenesis and progression (28, 29). Monocytes are released from the bone marrow, part of the tumor-derived secretome that can increase myelopoiesis and influence tumor proliferation, angiogenesis, and progression (30, 31). Lymphocytes are known to play an important role in tumor immune surveillance, induce cytotoxic cell death to defend against tumor cells, and inhibit tumor progression (IFN-γ) (32, 33). Simplistically synthesizing these, either increasing neutrophils and monocytes or decreasing lymphocytes, may disorder the immune balance, resulting in an elevation in the SIRI, which is correlated with a worse prognosis for gastric cancer patients.

The critical findings of this study, with a best cutoff value derived for SIRI, showed a statistical correlation of SIRI with DFS and OS: low SIRI, better survival; high SIRI, worse survival. That SIRI also correlated with Borrmann and pathologic stages is logical and further validates these findings. Finally, a comprehensive investigation of hematologic parameters in peripheral venous blood may help to discover new immunologic targets for individualized treatment.

### Conclusion

SIRI may qualify as a useful, reliable, and convenient prognostic indicator in patients with advanced gastric cancer to help physicians provide personalized prognostication for gastric cancer patients treated with NACT and surgical resection. Certainly, further studies are needed to verify the preliminary results of SIRI in larger groups of gastric cancer patients.

## Data Availability

The original contributions presented in the study are included in the article/Supplementary Material, further inquiries can be directed to the corresponding authors.

## Abbreviations

DFS, disease-free survival; GC, gastric cancer; L, lymphocyte; M, monocyte; MLR, monocyte to lymphocyte ratio; .N, neutrophil; NACT, neoadjuvant chemotherapy; NCI-CTC, National Cancer Institute Common Toxicity Criteria; NLR, neutrophil to lymphocyte ratio; OS, overall survival; P, platelet; PLR, platelet to lymphocyte ratio; RECIST, Response Evaluation Criteria In Solid Tumors; ROC, receiver operating characteristic curve; SIRI, systemic inflammation response index.
