# Supplementary material for: In Gastric Cancer Patients Receiving Neoadjuvant Chemotherapy Systemic Inflammation Response Index is a Useful Prognostic Indicator
Source: Pathol Oncol Res. 2021 Oct 12;27:1609811. doi: 10.3389/pore.2021.1609811 (PMC8546636; doi:10.3389/pore.2021.1609811)
Supplement: Supplementary file 1 [file Table1.docx]

Supplementary Table 1 Univariate and multivariate Cox proportional hazards regression model survival analysis of SIRI

|  |  | DFS |  |  |  | OS |  |  |
| --- | --- | --- | --- | --- | --- | --- | --- | --- |
| Parameters | Univariate analysis |  | Multivariate analysis |  | Univariate analysis |  | Multivariate analysis |  |
|  | Hazard ratio (95%CI) | *P* value | Hazard ratio (95%CI) | *P* value | Hazard ratio (95%CI) | *P* value | Hazard ratio (95%CI) | *P* value |
| Age (<56y / >=56y) | 1 / 5.809 (2.301-14.666) | ＜0.001 | 1 / 2.044 (1.069-3.912) | 0.031 | 1 / 4.617 (1.831-11.642) | ＜0.001 | 1 / 1.870 (1.014-3.546) | 0.034 |
| Gender (Male / Female) | 1 / 0.760 (0.250-2.310) | 0.628 |  |  | 1 / 0.705 (0.226-2.194) | 0.546 |  |  |
| BMI (<22.10 / >=22.10) | 1 / 1.947 (0.779-4.886) | 0.154 |  |  | 1 / 1.532 (0.843-2.782) | 0.161 |  |  |
| ABO blood type (A+B+AB / O) | 1 / 0.974 (0.364-2.606) | 0.958 |  |  | 1 / 1.185 (0.437-3.214) | 0.739 |  |  |
| NACT regimens (SOX+XELOX / Others) | 1 / 0.734 (0.148-3.636) | 0.705 |  |  | 1 / 0.421 (0.081-2.183) | 0.303 |  |  |
| Preoperative chemotherapy times (<3 / >=3) | 1 / 5.595 (1.954-16.018) | 0.001 | 1 / 2.302 (1.105-4.795) | 0.026 | 1 / 3.929(1.430-10.794) | 0.008 | 1 / 2.183 (1.062-4.487) | 0.034 |
| Postoperative chemotherapy regimens (SOX+XELOX / Others / No) | 1 / 3.936 (0.921-16.810) / 0.359 (0.014-9.210) | 0.100 |  |  | 1 / 1.611(0.402-6.461) / 0.169(0.007-4.031) | 0.367 |  |  |
| Postoperative chemotherapy times (0 / <4 / >=4) | 1 / 0.090 (0.004-0.647) / 0.191 (0.065-0.560) | ＜0.001 | 1 / 0.284 (0.121-0.666) / 0.479 (0.266-0.864) | 0.002 | 1 / 0.045(0.003-0.795) / 0.291 (0.098-0.863) | 0.001 | 1 / 0.269 (0.113-0.637) / 0.337 (0.190-0.559) | 0.005 |
| Radical resection (R0 / R1+R2) | 1 / 9.265 (3.812-22.517) | ＜0.001 | 1 / 3.190 (1.810-5.624) | ＜0.001 | 1 / 10.867(4.328-27.287) | ＜0.001 | 1 / 3.926 (2.267-6.800) | ＜0.001 |
| Type of surgery (distal gastrectomy / proximal and total gastrectomy) | 1 / 0.937 (0.262-3.344) | 0.920 |  |  | 1 / 1.067 (0.295-3.857) | 0.922 |  |  |
| Differentiation (poorly / moderately and well differentiated) | 1 / 0.830 (0.460-1.498) | 0.536 |  | \ | 1 / 0.637(0.305-1.329) | 0.229 |  |  |
| Primary tumor site (upper 1/3 / middle 1/3 / low 1/3) | 1 / 6.328 (0.995-40.231) / 2.336 (0.567-9.628) | 0.147 |  |  | 1 / 4.601 (0.688-30.748) / 2.451 (0.584-10.276) | 0.363 |  |  |
| Pathology (normal (Tis) and adenocarcinoma / mucinous and signet ring cell and mixed carcinoma) | 1 / 9.374 (2.568-34.212) | 0.001 | 1 / 1.981 (1.165-3.369) | 0.012 | 1 / 5.868(1.666-20.672) | 0.006 | 1 / 1.683 (1.093-3.096) | 0.043 |
| Clinical TNM classification |  |  |  |  |  |  |  |  |
| T stage (T3 / T4) | 1 / 2.626 (0.351-19.631) | 0.347 |  |  | 1 / 1.901 (0.254-14.208) | 0.531 |  |  |
| N stage (N0 / N1+N2) | 1 / 3.637 (1.114-11.875) | 0.032 | 1 / 2.304 (1.351-4.167) | 0.002 | 1 / 3.973 (1.181-13.373) | 0.026 | 1 / 4.151 (1.978-8.710) | ＜0.001 |
| TNM stage (II / III) | 1 / 2.375 (0.667-8.450) | 0.181 |  |  | 1 / 3.220(0.874-11.866) | 0.079 |  |  |
| Pathological TNM classification |  |  |  |  |  |  |  |  |
| T stage (Tis+T1 / T2+T3+T4) | 1 / 18.711 (2.711-129.153) | 0.002 | 1 / 9.797 (2.255-42.551) | 0.002 | 1 / 20.266 (3.132-131.156) | 0.002 | 1 / 12.219 (2.844-52.499) | ＜0.001 |
| N stage (N0 / N1+N2+N3) | 1 / 11.890 (2.393-59.134) | 0.002 | 1 / 2.955 (1.035-8.433) | ＜0.001 | 1 / 23.700 (4.133-135.921) | ＜0.001 | 1 / 4.123 (1.365-12.450) | 0.012 |
| Metastasis (M0 / M1) | 1 / 3.219 (0.437-23.736) | 0.251 |  |  | 1 / 1.122 (0.164-7.660) | 0.906 |  |  |
| TNM stage (Tis+I / II+III+IV) | 1 / 1.591 (0.045-55.672) | 0.798 |  |  | 1 / 1.260 (0.031-50.844) | 0.903 |  |  |
| Total lymph nodes (<27 / >=27) | 1 / 1.456 (0.593-3.576) | 0.412 |  |  | 1 / 1.334 (0.517-3.440) | 0.551 |  |  |
| Positive lymph nodes (<3 / >=3) | 1 / 4.851 (1.742-13.513) | 0.003 | 1 / 2.869 (1.636-5.032) | ＜0.001 | 1 / 3.466(1.292-9.296) | 0.014 | 1 / 2.987 (1.738-5.135) | ＜0.001 |
| Lauren classification (Intestinal / Diffuse / Mixed) | 1 / 1.034 (0.496-2.153) / 1.063 (0.473-2.387) | 0.989 |  |  | 1 / 1.656 (0.601-4.563) / 1.385 (0.584-3.285) | 0.620 |  |  |
| Borrmann classification (I+II / III+IV) | 1 / 2.641 (1.142-6.108) | 0.023 | 1 / 3.209 (1.565-6.577) | ＜0.001 | 1 / 2.618 (1.149-5.963) | 0.022 | 1 / 3.102 (1.542-6.239) | ＜0.001 |
| Tumor size (<50 / >=50mm) | 1 / 2.669 (1.264-5.638) | 0.010 | 1 / 1.784 (1.018-3.242) | 0.037 | 1 / 2.635(1.259-5.514) | 0.010 | 1 / 1.916 (1.048-3.500) | 0.035 |
| White blood cell (<6.42 / >=6.42) | 1 / 2.040 (0.861-4.834) | 0.105 |  |  | 1 / 1.633 (0.849-3.141) | 0.141 |  |  |
| Hemoglobin (<121 / >=121) | 1 / 2.015 (0.866-4.683) | 0.103 |  |  | 1 / 1.903(0.839-4.316) | 0.123 |  |  |
| Neutrophils (<3.82 / >=3.82) | 1 / 2.120 (0.645-6.968) | 0.216 |  |  | 1 / 1.728(0.494-6.037) | 0.392 |  |  |
| Monocyte (<0.45 / >=0.45) | 1 / 1.863 (0.920-3.769) | 0.084 |  |  | 1 / 2.461 (0.838-7.225) | 0.101 |  |  |
| Platelet (<285 / >=285) | 1 / 2.593 (0.857-7.844) | 0.092 |  |  | 1 / 2.548(0.810-8.014) | 0.110 |  |  |
| Lymphocyte (<1.72 / >=1.72) | 1 / 0.249 (0.085-0.732) | 0.012 | 1 / 0.450 (0.215-0.942) | 0.034 | 1 / 0.281 (0.097-0.816) | 0.020 | 1 / 0.472 (0.222-0.993) | 0.022 |
| NLR (<2.18 / >=2.18) | 1 / 2.092 (0.730-5.995) | 0.169 |  |  | 1 / 1.569 (0.558-4.408) | 0.393 |  |  |
| MLR (<0.28 / >=0.28) | 1 / 1.147 (0.290-4.536) | 0.845 |  |  | 1 / 1.772 (0.431-7.274) | 0.427 |  |  |
| PLR (<164 / >=164) | 1 / 0.787 (0.235-2.635) | 0.697 |  |  | 1 / 0.754 (0.215-2.644) | 0.659 |  |  |
| SIRI (<1.21 / >=1.21) | 1 / 3.437 (1.059-11.149) | 0.009 | 1 / 1.782 (1.241-3.942) | 0.024 | 1 / 3.331(1.001-11.082) | 0.021 | 1 / 1.665 (1.302-3.613) | 0.028 |
